# Disease awareness or subtle product placement? Orphan diseases featured in the television series “House, M.D.” - a cross-sectional analysis

**DOI:** 10.1186/s12910-020-0463-x

**Published:** 2020-03-14

**Authors:** Konstantin Mechler, Juliane Rausch, William K. Mountford, Markus Ries

**Affiliations:** 1 Department of Addictive Behavior and Addiction Medicine, Central Institute of Mental Health, Medical Faculty Mannheim, University of Heidelberg, Mannheim, Germany; 2 Department of Child and Adolescent Psychiatry and Psychotherapy, Central Institute of Mental Health, Medical Faculty Mannheim, University of Heidelberg, Mannheim, Germany; 3 University of North Carolina, Wilmington, NC USA; 4 Allergan plc., Madison, NJ USA; 5 Division of Pediatric Neurology and Metabolic Medicine, Center for Rare Diseases, Center for Pediatric and Adolescent Medicine, University Hospital Heidelberg, INF 430, 69120 Heidelberg, Germany; 6 Center for Rare Diseases, University Hospital Heidelberg, INF 430, 69120 Heidelberg, Germany

**Keywords:** Orphan diseases, Rare disease, Orphan drugs, Advertisement, Public awareness

## Abstract

**Background:**

Approximately 7% of the general population is affected by an orphan disease, which, in the United States, is defined as affecting fewer than 1 in 1500 people. Disease awareness is often low and time-to-diagnosis delayed. Different legislations worldwide have created incentives for pharmaceutical companies to develop drugs for orphan diseases. A journalistic article in *Bloomberg Businessweek* has claimed that pharmaceutical companies have tried marketing orphan drugs by placing a specific disease into the popular television series “House, M.D.” which features diagnostic journeys and was produced between 2004 and 2012. This study aimed to describe the presentation of orphan diseases in the television series “House, M.D.”, to test in an exploratory fashion the hypothesis that treatable orphan conditions are overrepresented in “House, M.D.” and to discuss whether such marketing practices may or may not be ethical.

**Methods:**

A list of all medical cases depicted in the television series “House, M.D.” was obtained and classified as orphan or non-orphan according to the Orphanet database. The ratios of orphan diseases among all diseases, such with an orphan drug designation and such with an orphan drug approval by the FDA were then compared with conservative approximations of real world conditions (chi-squared tests for equality of proportions). STROBE criteria were respected.

**Results:**

Out of a total of *n* = 181 different medical diagnoses, *n* = 42 (23.2%) were orphan diseases. The difference in percentages in between “House, M.D.” and reality was not statistically significant for orphan diseases overall (*p* = 0.96), yet was statistically significantly higher for both orphan diseases with one or more orphan drug designations (*p* = 0.0192) and such with one or more approved orphan drugs (*p* < 0.0001).

**Conclusions:**

Orphan diseases with a designated and/or approved orphan drug were overrepresented in the television series “House, M.D.” with statistical significance while orphan diseases overall were not. This may be explained by (so far) undocumented efforts of pharmaceutical companies to place their orphan drugs in the television series, as described in the article in *Bloomberg Businessweek*. Further research is needed into marketing practices in popular and emerging media formats.

## Introduction

The *Rare Diseases Act of 2002* by the United States Congress defines orphan diseases according to their prevalence as affecting less than one person per 1500 in the population [1]. Approximately 7000 orphan diseases have been described and 6 to 8% of the general population are affected by an orphan disease, among these around 25 million patients in the US and 30 million patients in the European Union [2, 3]. Orphan diseases are in general chronic conditions and show high degrees of morbidity and mortality. Treatment options may not be available at all and making the diagnosis of disease may be delayed because the respective orphan disease is not always included in differential diagnostic considerations [4–7]. The relatively long time between the appearance of first signs or symptoms of an orphan condition and the establishment of the correct diagnosis is presumably due to low disease awareness as recently demonstrated in conditions such as molybdenum cofactor deficiency, mucopolysaccharidosis type VII, and Farber disease [8–10]. Timely diagnosis becomes even more crucial once there is a specific therapy available for a disease that is irreversibly progressive if untreated. In addition, there is a monetary aspect towards disease awareness:

As pharmaceutical companies work in a value- and profit-oriented way, diseases that only affect very few people do not per se represent lucrative targets for drug development because the volume of sales is substantially limited. Different legislative bodies worldwide such as the US (*Orphan Drug Act of 1983*) and the EU (*Regulation No. 141* in 2000), have passed acts intended to address these challenges by adjusting the regulatory framework of “traditional” drug development and providing an opportunity to make orphan drug development a profitable venture for pharmaceutical companies [11]. In the US, the *Orphan Drug Act of 1983* implemented various incentives, such as 7-years’ marketing exclusivity, tax credit for 50% of clinical trial costs, protocol assistance, fee waiver at the US Food and Drug Administration (FDA), and qualification for the orphan products grants program [11]. The regulatory path to approval of an orphan drug starts with an “orphan drug designation” by the regulatory body (the FDA in the US) which confirms the qualification of the respective drug for the above-mentioned benefits and enables the sponsor to investigate the drug’s effectiveness and tolerability in clinical trials. This may ultimately lead to an orphan drug approval. At the US Food and Drug Administration (FDA) between January 1983 and May 2015, 3425 orphan drug designations and 492 orphan drug approvals had been granted [12]. At the European Medicines Agency (EMA), 845 applications for orphan drug designations had been filed between 2000 and 2010, 80.9% of which were granted. In the same period 108 marketing approvals were applied for, of which 63 (58%) were granted [13]. By June 2012, a total of 70 orphan drugs had been approved in the EU [14]. The most common disease group to be treated with these orphan drugs has been found to be malignancies [15]. Data from 2010 shows that 60% of all FDA approved orphan drugs were developed by large or established pharmaceutical companies while 38% were developed by small and medium pharmaceutical companies and only 2% by academic institutes [16]. The interest of larger pharmaceutical companies to explore and invest in the orphan drug sector is shown by figures made available by the European Federation of Pharmaceutical Industries and Associations. When comparing the sectors of research and development investment, the sector “Rare Diseases” ranks highest with 24.6% of total investments, followed by “Pharmaceuticals” (15.9%) and “Software and Computers” (9.8%) [17]. Two advantages of orphan drugs compared to non-orphan drugs have been scientifically analyzed and may, among others, be responsible for these investments: orphan drugs show a significantly shorter development time (4 years vs. 5.5 years from phase II trials to launch date) and a larger probability of successful approval (93% vs. 88%). Both differences between orphan and non-orphan drugs were statistically significant [18].

An article in *Bloomberg Businessweek* has claimed that pharmaceutical companies have tried placing specific orphan diseases into the popular television series “House, M.D.”. The article did not cite any sources or facts further supporting this claim. “House, M.D.” was produced in the United States in between 2004 and 2012 and spanned 177 episodes over eight seasons. Each episode’s plot of this show features a diagnostic journey of a given patient [19]. Such “product placement” in “House, M.D.” could have meant an immensely effective form of advertisement because with a maximum average viewership of 19.4 million per episode a broad audience is being reached.

The aim of this study was therefore to describe the presentation of orphan diseases in the television series “House, M.D.”, to test in an exploratory fashion the hypothesis that treatable orphan conditions are overrepresented in the pattern of diseases depicted in the televisions series “House, M.D.” and to discuss whether such marketing practices may or may not be ethical.

## Material and methods

This study was designed, executed, and analyzed, taking into account the principles outlined in the Strengthening the Reporting of Observational Studies in Epidemiology Statement (STROBE).

A list of all medical cases depicted in the television series “House, M.D.” was obtained from the so-called “House Wiki”, an online database created and operated by fans of the television series [20]. Diagnoses were classified as orphan or non-orphan according to the Orphanet database accessible at http://www.orpha.net/. Orphanet is an international initiative by academic institutions from 37 countries and and led by the Orphanet Coordinating team at the French National Institute of Health and Medical Research (INSERM) in Paris. Its primary mission is to provide a comprehensive and easily accessible inventory of orphan diseases and orphan drugs and enhance visibility and knowledge on orphan diseases.

Furthermore for each orphan disease depicted in “House, M.D.”, the ‘Orphan Drug Product designation database’ provided by the FDA online was searched for potential orphan drug designations and/or approvals granted by the FDA [21]. The FDA was chosen because “House, M.D.” was produced in the United States. Download of data occurred on April 3rd 2018.

In order to test in an exploratory fashion the hypothesis that treatable orphan conditions are overrepresented in the pattern of diseases depicted in the televisions series “House, M.D.” a comparison with real-world data was performed. The ratio of orphan diseases among all known diseases was approximated on basis of figures by the World Health Organization (WHO), which has estimated that out of 30,000 diseases overall approximately 7000 are defined as orphan [2, 3, 22–25]. The resulting real-world ratio of orphan among all diseases is therefore 23%. This ratio was compared with the corresponding ratio determined in “House, M.D.”. Furthermore, the rates of orphan diseases with one or more orphan drug designation for diseases presented in “House, M.D.” were compared with the corresponding rates provided by the FDA online database. The same was applied for approvals.

Since the commencement of the US *Orphan Drug Act of 1983* until the close of database on April 3rd 2018, 4527 drugs for 2762 orphan diseases had been granted an orphan drug designation and 676 drugs for 534 orphan diseases had received an orphan drug approval by the FDA in the United States [21]. With approximately 7000 orphan diseases in existence, this leads to a ratio of 39.5% for diseases with orphan drug designations vs. overall orphan diseases (2762 out of 7000) and 7.6% for diseases with orphan drug approvals vs. overall orphan diseases (534 out of 7000). Multiple orphan drug designations and/or approvals were omitted.

### Statistical analyses

Standard methods of descriptive statistics were applied. Furthermore, rates of orphan diseases and approved orphan drug were compared using chi-squared tests with Yate’s correction. *P*-values reported are two-sided. P-values of 0.05 or less were deemed statistically significant. Data were collected in Microsoft® Office Excel. All analyses were performed with IBM® SPSS® Statistics Version 23.

## Results

A total of *n* = 181 different medical diagnoses have been depicted in “House, M.D.” during all 117 episodes spanning 8 seasons between 2004 and 2012. Of these, *n* = 42 (23.2%) were orphan diseases. This ratio was only slightly higher than the estimated 23% in overall medicine and the comparison was not statistically significant (*p* = 0.96, chi-squared test with Yate’s correction). Twenty-five orphan diseases, i.e. 58.1% of the orphan conditions depicted in “House, M.D.” had one or more FDA orphan drug designations. The comparison of this ratio with the real-world ratio (39.5% of all known orphan diseases) was statistically significant (*p* = 0.0192, chi-squared test with Yate’s correction). Fifteen diseases, i.e. 39.4% of the orphan conditions depicted in “House, M.D.” had one or more orphan drug approvals. The comparison of this ratio with the real-world ratio Fig. (7.6% of orphan diseases) was also statistically significant (*p* < 0.0001, chi-squared test with Yate’s correction). Table 1 gives an overview of the results. Table 2 shows all presented orphan diseases and corresponding pharmaceutical compounds with an orphan drug designation, if present, as listed in the Orphanet database. Of note, one orphan disease (leprosy) was depicted twice (season 1, episode 13 and season 5, episode 1).

**Table 1:** Overview of results

	Real-world	“House, M.D.”	Comparison of real-world versus “House, M.D.” *
All diseases (n)	30,000	181	
Orphan diseases overall (n)	7000	42	
Ratio of orphan diseases among all diseases	23%	23.2%	*p* = 0.96
Orphan diseases with orphan drug approval	534	15	
Ratio of orphan diseases with orphan drug approval among all orphan diseases	7.6%	39.4%	***p = 0.0192***

*p-value from chi-squared test with Yate’s correction

**Table 2:** Orphan diseases presented on the television series “House, M.D.” and corresponding orphan drug designations and approvals by the FDA, if present, as well as respective season and episodes

Orphan disease	ORPHA Number	Number of compounds with an orphan drug designation (by the FDA)	Number of compounds with an orphan drug approval (by the FDA)	FDA approved compounds	Season	Episode
Sickle cell trait	232	40	3	Hydroxyurea, L-glutamine	7	2
Mastocytosis	98,292	8	3	Cromolyn sodium, imatinib mesylate, midostaurin	8	1
Muckle-Wells syndrome	575	3	3	Anakinra, Canakinumab, Rilonacept	7	14
Ornithine Transcarbamylase Deficiency	664	4	2	Benzoate and phenylacetate, sodium phenylbutyrate	1	15
Familial Mediterranean fever	342	3	2	Canakinumab, colchicine	5	6
Fabry disease	324	9	1	Ceramide trihexosidase/alpha-galactosidase A	6	3
African trypanosomiasis	3385	4	1	Eflornithine HCl	1	7
Acute intermittent porphyria	79,276	4	1	Hemin	1	22
Leprosy	548	3	1	Clofazimine	1	13
Diffuse lepromatous leprosy	548	3	1	Clofazimine	5	1
Wegener’s disease	900	3	1	Rituximab	7	23
Chronic granulomatous disease	379	2	1	Interferon gamma 1-b	3	8
Mucormycosis	73,263	2	1	Isavuconazonium sulfate	8	14
Hereditary coproporphyria	79,273	2	1	Hemin	5	10
Hereditary Angioedema	91,378	2	1	C1-esterase-inhibitor	3	5
Thrombotic thrombocytopenic purpura	54,057	7	0	–	1	19
Hereditary hemorrhagic telangiectasia	774	3	0	–	3	16
Senile amyloidosis	330,001	3	0	–	3	3
Alport syndrome	63	2	0	–	8	7
Giant cell arteritis	397	1	0	–	8	12
Von Hippel-Lindau syndrome	892	1	0	–	4	2
Wiskott-Aldrich Syndrome	906	1	0	–	5	15
Subacute sclerosing panencephalitis	2806	1	0	–	1	2
Variegate porphyria	79,473	1	0	–	7	10
Ehlers-Danlos Syndrome	98,249	1	0	–	7	18
Langerhans cell histiocytosis	389	0	0	–	3	10
MERRF syndrome	551	0	0	–	3	7
Henoch-Schönlein Purpura	761	0	0	–	6	18
Adult Refsum disease	773	0	0	–	7	17
Kawasaki’s syndrome	2331	0	0	–	8	5
Reye’s syndrome	3096	0	0	–	8	9
Takayasu’s arteritis	3287	0	0	–	3	15
Whipple’s disease	3452	0	0	–	6	15
Bartonella	50,839	0	0	–	7	16
McLeod syndrome	59,306	0	0	–	7	12
Rickettsialpox	83,312	0	0	–	7	7
Miller Fisher syndrome	98,919	0	0	–	8	16
Polyglandular autoimmune syndrome type III	227,982	0	0	–	8	13
Hughes-Stovin syndrome	228,116	0	0	–	6	11
Marburg multiple sclerosis	228,157	0	0	–	7	8
Arnold-Chiari malformation	268,882	0	0	–	6	19
Primary antiphospholipid syndrome	398,097	0	0	–	6	5

## Discussion

Orphan diseases accounted for roughly a quarter of all medical cases depicted in the television series “House, M.D.” which reflects the proportion of orphan diseases among all known diseases in the general population. In contrast, both the ratios of orphan diseases with an orphan drug designation and with an approved orphan drug depicted in “House, M.D.” were statistically significantly higher than the ratios expected from the estimated natural epidemiology. This indicates that treatable orphan conditions or orphan diseases with a therapeutic compound in development are overrepresented in the television series “House, M.D.”. The precise process for this phenomenon remains speculative.

While these results further substantiate the claim raised by the article in *Bloomberg Businessweek* it also mandates very careful interpretation since different reasons may account for these findings. The rare and arcane nature of such diseases suits a fictional television (TV) series which builds on suspense, excitement and a detective-like main character portrayed as a weird yet excellent diagnostician. “House, M.D.” was an extremely popular television series across the world. In its third season in the US, the series had an average viewership of 19.4 million per episode [26]. It was reported to have reached 81.8 million people in 66 different countries and was especially popular among physicians in training [27]. The series has both been applauded and criticized for its medical and scientific accuracy – especially since many presented cases were considered to be very unlikely, yet not impossible. Despite the appeal of orphan diseases in general, this study found that orphan diseases themselves were not overrepresented in “House, M.D.”. Yet, those with a designated and/or approved orphan drug were.

The findings of this study may be explained by (so far) undocumented efforts of pharmaceutical companies to place their orphan drugs in the TV series, as described in the article in *Bloomberg Businessweek*. Despite all the epidemiological, scientific and methodological challenges, orphan drug development and marketing is considered a viable business model for the pharmaceutical industry [28].

It does not seem surprising that orphan drugs and orphan diseases are featured in TV series since pharmaceutical marketing and health care communication has become a multimodal, holistic approach which incorporates a variety of communication platforms including audiovisual media as recently illustrated by Sponder and Mattingley [29, 30]. Advertising / marketing of pharmaceutical products is generally limited and regulated worldwide, albeit with significant international differences. Direct to consumer advertising (DTCA) of prescription drugs is only legal in the US and New Zealand [31]. In the US, the FDA regulates DTCA with certain limits and requirements regarding the content and presentation. The ban of DTCA in other countries has led to a rise in disease awareness advertising (DAA) where not the prescription drug itself but the disease it is intended to treat is promoted instead, e.g. by awareness campaigns providing information about diagnosis and treatment options [31]. Critics have described DAA going as far as “disease mongering”, which Moynihan et al. describe as “widening the boundaries of treatable illness in order to expand markets for those who sell and deliver treatments” [32].

The potential effects of both DTCA and DAA are a current point of discussion. Benefits may include increased disease awareness, higher diagnostic rates and ultimately better treatment with a higher adherence to treatment as well as informed and “empowered” patients. Negative effects may be unbalanced information which overtaxes consumers, overdiagnosis and overtreatment [33, 34]. The limited research evidence confirms the existence of both positive and negative effects with a tendency towards larger negative effects overall [34, 35].

To the authors’ knowledge, no orphan drug was specifically mentioned or presented in any of the episodes of “House, M.D.”. Consequently, an intentional placement of orphan diseases in the series, brought forward by the Bloomberg Businessweek article, may be considered as DAA. Its legality in the US and other countries in which “House, M.D.” airs/aired may be questionable depending on the local laws and jurisdiction. In the US, pharmaceutical companies have been prosecuted in the past for false advertising, e.g. miscommunication regarding side effects [36, 37].

Aside from the aspect of legality, such non-transparent product placement may be questionable from an ethical view. The viewership is unaware of being targeted with information intended to advertise products. Vulnerable groups such as children and young adults remain unprotected, e.g., when advertising alcohol products [38]. Product placement of non-pharmaceutical products such as cars and electronic devices has increased in frequency and intensity over the last decades, especially in US (Hollywood) movies and television formats [39].

But there may also be benefits that can be weighed against such concerns. Despite the relatively high prevalence of all orphan diseases combined (see above), awareness of these in the population and medical and administrative professionals is still an issue. Low disease awareness leads to diagnostic delay, and the time between onset of disease and diagnosis can be substantial in some orphan diseases [8–10]. This is associated with uncertainty for the afflicted patients and families. In addition, appropriate disease management and timely treatment may be delayed. The positive effect of media presence for orphan disease is that disease awareness may improve and time to diagnosis may therefore decrease in the future. Overall, the portrayal of such diseases and diagnostic journeys in an entertainment-focused setting such as “House, M.D.” could have beneficial effects for the orphan disease community as a whole as general awareness is raised for this important group of diseases. Yet, it seems necessary to disclose such product placement to the viewer in a transparent way (e.g., visible during opening and end credits).

In order to improve the current situation, it seems desirable to achieve more transparency about industry involvement and financial support, especially in advertisement campaigns. This is echoed by the World Health Organization (WHO) which has called for a review and update of the WHO’s criteria for ethical drug promotion [40]. Furthermore, disease awareness campaigns funded by public or primarily non-company sources could and should be increased. A prominent example is the “Rare Disease Day*”* which takes place on the last day of February each year since 2008. It is organized by EURORDIS (Rare Diseases Europe), a non-profit initiative of 869 orphan disease patient organisations from 71 countries. Of interest, EURORDIS is funded by patient organizations (36%), health sector corporates (27%), the European Commission (26%), event fees (5%) and other sources (6%). Another interesting related development is the recent use of so called “non-profit product placement” by non-governmental organizations, e.g. Amnesty International, to promote their cause [41].

Orphan drug pricing is currently a subject of major public debate with particular relevance for ethical concerns [42–44]. An example for this is the orphan drug *Eteplirsen* which received accelerated approval by the FDA for treatment Duchenne Muscular Dystrophy in 2016 [45, 46]. This decision and the clinical benefit of *Eteplirsen* had by itself been widely questioned, when major discussion arose about the estimated treatment costs of $300,000 to 400,000 per year per patient [47]. Depending on the health care system in the respective country, such high drug prices necessarily limit access to such drugs which is certainly highly questionable from an ethical view [47]. *Eteplirsen* also functions as an example of the ethical consideration whether a high drug price, which may also enlarge expectations by patients, is reasonable when only little clinical benefit results. In general, the relatively high price of orphan drugs could be another reason that the pharmaceutical industry would wish to increase public awareness of orphan diseases.

The present study has several limitations. First, the data presented and their interpretation cannot prove causal relationships. Second, more prevalent orphan diseases may be more likely to be depicted in a TV series. Previous literature has shown that an orphan drug development is more likely in the more prevalent orphan diseases [48]. Third, the real benefit of such “product placement” is questionable as orphan drugs will only be of therapeutic interest for a very small portion of the television series’ viewership. Fourth, the number of overall diseases and overall orphan diseases are estimations which may lead to some uncertainty in the generalizability of the findings.

## Conclusions

Orphan diseases with a designated and/or approved orphan drug were statistically significantly overrepresented in the television series “House, M.D.” compared with the expected ratio deducted from the natural epidemiological distribution of diseases. This may be explained by (so far) undocumented efforts of pharmaceutical companies to place their orphan drugs in the TV series, as described in the article in *Bloomberg Businessweek*. Further research is needed into marketing practices in popular and emerging media formats.

## Data Availability

The datasets analysed during the current study were extracted from three publicly available sources. First, all medical diagnoses depicted in “House, M.D.” were collected from the “House Wiki” (http://house.wikia.com/wiki/List_of_medical_diagnoses). Second, all featured diseases were then specifically searched for in the Orphanet database to obtain whether they were classified as orphan or non-orphan (https://www.orpha.net/consor/cgi-bin/Disease.php). Third, the existence of compounds with an orphan drug designation and/or approval by the FDA was recorded by searching the Orphan Drug Product designation database by the FDA for each specific orphan disease (https://www.accessdata.fda.gov/scripts/opdlisting/oopd/index.cfm).

## Abbreviations

DAA: Disease awareness advertising
DTCA: Direct to consumer advertising
EMA: European Medicines Agency
EU: European Union
EURORDIS: Rare Diseases Europe
FDA: United States Food and Drug Administration
IBM: International Business Machines Corporation
INSERM: French National Institute of Health and Medical Research
M.D.: Medicinae Doctor/Doctor of Medicine
SPSS: Statistical Package for the Social Sciences
STROBE: Strengthening the Reporting of Observational Studies in Epidemiology Statement
TV: Television
US: United States
WHO: World Health Organization
